# British Health Stations.—V

**Published:** 1905-06-10

**Authors:** 


### BRITISH HEALTH STATIONS.—V.

[By our Special Medical Commissioner.]

### ST. IVES, CORNWALL.

Among the quaint, picturesque and peaceful havens of the "Delectable Duchy," St. Ives merits a foremost place. This ancient little town, the home of artist and fisherman and the resort of antiquary and student, is an ideal health station. It is perhaps the most interesting of the many charming spots on the Cornish Riviera. The little town is situated on the northern coast of Cornwall along the glorious sweep of St. Ives Bay and spreads out along a narrow isthmus to a small promontory which faces the roll of the Atlantic breakers. This ancient home of the sturdy fisher-folk affords endless charm to the visitor.*

The grey, weather-beaten, irregularly-placed houses cluster along the sides of narrow, twisting, stone-paved lanes and present an old-world appearance which has held captive many an artist. The St. Ives school of artists has now won world-wide distinction. During the fishing season the centre of all interests focus in the ancient harbour. Here the toil-worn and honest fishermen and the home-abiding, simple, patient wives congregate, and live their hard, but peaceful, lives regardless of the coming and going of the fleeting but ever welcome visitor.

St. Ives can provide for almost all the requirements of the health-seeker needing rest of mind, quiet for the weary body, shelter from trying winds, invigorating breezes, much sunlight, and a climate which, while warm and equable, is not enervating.

Dr. J. M. Nicholls, the medical officer of health, informs us that the death-rate is only 14·18. The water supply has been improved and good progress is being made in providing all sanitary requirements.

The mean temperature is given as 52·22°. The rainfall is returned as 39·51 inches. Days of sunshine total 289. During the winter the mean temperature is said to be 53·5°, with a mean daily range of 8·4°.†

The prevailing winds are westerly and north-westerly, and are bracing and rich in buoyant power. The high land above the town affords protection and also provides a stimulating and invigorating upland for walkers.

St. Ives is an admirable centre, from whence the Land's End, Lizard district, and other places of interest to the naturalist and artist, as well as to the visitor and health-seeker, can be easily reached.

There are many opportunities for such health-bringing sports and pastimes as fishing, sailing, bathing, driving, cycling, and the golf links close by at Lelant are justly celebrated.

There is good accommodation in the way of comfortable lodgings, well-managed boarding-houses and hotels. Special mention must be made of the old family mansion of Tregenna Castle, which the Great Western Railway Company, with rare wisdom and artistic discernment, has converted into a hotel with all the comforts and conveniences of a modern establishment, yet without in any way sacrificing the quiet charm, delightful seclusion and homelike appearance of a private country house.

We have stayed at Tregenna Castle and consider it an admirable haven of rest for the overworked and jaded professional man. Here may be found all necessary requirements for recuperation of body and recreation of mind.

St. Ives, especially in springtime and during early summer days, is a perfect health resort for the artistic invalid and nature-loving convalescent. Doctors and nurses, and all persons feeling the result of the stress and strain of winter work in busy centres, should pilgrimage to the health-giving shrine of Saint Ives.‡

By the enterprise of the Great Western Railway this western resort has been brought near. Although 324 miles distant from Paddington it can be reached in a little over seven hours, the journey being rendered comfortable and enjoyable by the many conveniences provided.

* See "Guide to Saint Ives," by J. H. Matthews." St. Ives: James Wearne. Good maps of district in "North Devon and North Cornwall," by C. S. Ward, M.A. Eighth edition. London: 1903.

† The Cornish Riviera. Issued by the Great Western Railway. London. 1904.

‡ A well-illustrated little Guide to St. Ives has recently been issued by W. and J. Jacobs, of Fore Street, St. Ives; and may well be consulted by intending visitors.

